# Microbial Diversity and Biochemical Analysis of Suanzhou: A Traditional Chinese Fermented Cereal Gruel

**DOI:** 10.3389/fmicb.2016.01311

**Published:** 2016-08-25

**Authors:** Huibin Qin, Qinghui Sun, Xuewei Pan, Zhijun Qiao, Hongjiang Yang

**Affiliations:** ^1^Key Laboratory of Industrial Microbiology, Ministry of Education, Tianjin Key Laboratory of Industrial Microbiology, College of Biotechnology, Tianjin University of Science and TechnologyTianjin, China; ^2^Key Laboratory of Crop Gene Resources and Germplasm Enhancement on Loess Plateau, Ministry of Agriculture, Shanxi Key Laboratory of Genetic Resources and Genetic Improvement of Minor Crops, Institute of Crop Germplasm Resources of Shanxi Academy of Agricultural SciencesTaiyuan, China

**Keywords:** Suanzhou, metagenomic analysis, lactic acid bacteria, acetic acid bacteria, yeast, free amino acid

## Abstract

Suanzhou as a traditional Chinese gruel is fermented from proso millet and millet. The biochemical analysis showed Suanzhou had relatively high concentrations of lactic acid, acetic acid, and free amino acids. The metagenomics of Suanzhou were studied, with the analysis of the V4 region of 16S rRNA gene, the genera *Lactobacillus* and *Acetobacter* were found dominant with the average abundance of 58.2 and 24.4%, respectively; and with the analysis of the ITS1 region between 18S and 5.8S rRNA genes, 97.3% of the fungal community was found belonging to the genus *Pichia* and 2.7% belonging to five other genera. Moreover, the isolates recovered from 59 Suanzhou samples with various media were identified with the 16S rRNA or 18S rRNA gene analyses. *Lactobacillus fermentum* (26.9%), *L. pentosus* (19.4%), *L. casei* (17.9%), and *L. brevis* (16.4%) were the four dominant *Lactobacillus* species; *Acetobacter lovaniensis* (38.1%), *A. syzygii* (16.7%), *A. okinawensis* (16.7%), and *A. indonesiensis* (11.9%) were the four dominant *Acetobacter* species; and *Pichia kudriavzevii* (55.8%) and *Galactomyces geotrichum* (23.1%) were the two dominant fungal species. Additionally, *L. pentosus* p28-c and *L. casei* h28-c1 were selected for the fermentations mimicking the natural process. Collectively, our data demonstrate that Suanzhou is a nutritional food high in free amino acids and organic acids. Diverse *Lactobacillus, Acetobacter*, and yeast species are identified as the dominant microorganisms in Suanzhou. The isolated strains can be further characterized and used as starters for the industrial production of Suanzhou safely.

## Introduction

Many types of ethnic fermented cereal foods are widely consumed across the world. Compared with foods cooked directly from raw materials, fermented cereal foods are generally more tasteful, easily digested, and richer in various nutrients, such as vitamins, organic acids, and free amino acids (Blandino et al., 2003). Almost all types of cereals have been prepared into many kinds of foods in various natural fermented processes. Diverse microorganisms, mainly comprised of a number of bacteria and yeast species originated from the cereal grains and local environments, have been identified with diverse techniques (Tamang et al., 2016a,b).

A number of indigenous fermented foods have been made of rice or rice as the main material, such as Idli, dominant with *Leuconostoc lactis* (Saravanan and Shetty, 2016); Ang-kak also named Chinese red rice, dominant with *Monascus* strains (Lotong and Suwanarit, 1990); Selroti, dominant with multiple LAB and yeast species (Das et al., 2012); and Jiuniang or Laozao, dominant with *Rhizopus, Mucor, Monilia, Aspergillus*, and yeast species (Li and Hsieh, 2004). Wheat is an important source of diet proteins. However, wheat-based foods may contain a certain level of gluten which may cause allergic reactions in some individuals. Fermented wheat flour foods can greatly reduce the gluten content to safe levels, such as Sourdough, dominant with *Lactobacillus* species, and *Saccharomyces cerevisiae* (Settanni et al., 2005); Bhatooru, dominant with *S. cerevisiae, Lactobacillus plantarum*, and *Bacillus* sp. (Savitri and Bhalla, 2013); and Miso, dominant with *Pediococcus acidilactici* (Asahara et al., 1992). Maize based fermented foods mainly include doklu, dominant with *Lactobacillus fermentum, L. plantarum*, and *Pediococcus pentosaceus* (Assohoun-Djeni et al., 2016); and Ogi, dominant with *P. acidilactici* and *Lactobacillus paraplantarum* (Okeke et al., 2015). Sorghum based fermented foods are consumed in a number of African countries, such as Injera (Fischer et al., 2014), Kisra (Mohammed et al., 1991), and Hussuwa (Yousif et al., 2010), and all of them are rich in lactic acid bacteria (LAB). Millet is another important cereal grain and consumed as a staple food throughout the world (Saleh et al., 2013). Dosa (Palanisamy et al., 2012) and Ben-saalga (Tou et al., 2006) are two types of fermented millet foods which are also rich in LAB.

Proso millet and millet are highly drought-resistant crops with low demanding to environments. In northwestern China, proso millet and millet are commonly fermented to make Suanzhou, a sour gruel food easily prepared in local individual households. Until now, no studies of Suanzhou have been conducted in terms of its nutrients and microbial populations. In this work, totally 59 Suanzhou samples were collected for metagenomic DNA analysis and detection of free amino acids and organic acids concentration. Additionally, the dominant microorganisms in Suanzhou were isolated, identified, and characterized for possible applications in industrial production of Suanzhou.

## Materials and methods

### Preparation of Suanzhou

Suanzhou is a gruel made of fermented cereals prepared in individual households. Briefly, four types of raw materials were used in fermentations, group A containing samples fermented from millet with a small amount of rice (<10%), group B from millet, group C from white proso millet, and group D from red proso millet (Table S1). About 100 g grains were soaked in the fermentation soup or supernatant from the previous fermentation and kept at room temperature for 24 h in a jar sealed with a lid. Fermented grains are taken away for cooking. Raw materials are again added and soaked in the acidic soup for future fermentation and consumption. The water loss is supplemented with boiled water. Thirty samples (h1-30) were from Hequ county, in which different proso millet were used (Table S1). Twenty-nine samples (p1-30, the sample p25 was contaminated and removed from the analyses) from Pianguan county, in which millet was used as main raw material (Table S1). Both counties are located in Shanxi Province, China. All the Suanzhou samples were collected after 24 h incubation. Two 50-ml sour soup samples were obtained from each jar-fermentor and stored at 4°C for assays.

### Metagenomic analysis of Suanzhou samples

The samples for metagenomic analysis were randomly selected on the basis of raw materials used for fermentation from two regions, Pianguan County and Hequ County. Suanzhou samples were centrifuged and the pellets were subjected to the extraction of genomic DNA by using Qiagen DNA blood and tissue kit (Qiagen, Dutch). To investigate the bacterial communities, the hypervariable V4 region (~207 bp) of the 16S rRNA gene was analyzed with the primers 520-F (5′ AYTGGGYDTAAA GNG 3′) and 802-R (5′ TACNVGGGTATC TAATCC 3′) (Cole et al., 2005). To investigate the fungal communities, the ITS1 region between 18S and 5.8S rRNA genes was analyzed with the primers ITS1-F (5′ CTTGGTCATTTAGAG GAAGTAA 3′) and ITS2 (5′ GCTGCGTTCTTC ATCGATGC 3′) (Schnabel et al., 1999). Metagenomic sequencing was performed on an Illumina MiSeq system by Shanghai Personal Biotechnology Co., Ltd., China (http://www.personalbio.cn). The obtained sequences were assigned to the operational taxonomic units (OTUs) with a threshold of 97% pairwise identity using the BLASTN tool in the NCBI (http://blast.ncbi.nlm.nih.gov/Blast.cgi). The species diversity, richness, and abundance were estimated by the Shannon, Chao1, and ACE indices (http://www.mothur.org/wiki/). Totally, 1,046,828 clean sequencing reads with length around 225 bp were obtained from the libraries of the 24 Suanzhou samples. Venn diagram was used to group the samples on basis of the genus level (http://bioinformatics.psb.ugent.be/webtools/Venn/). The possible correlations of the microbial communities were analyzed by the online software Cytoscape (www.mothur.org/wiki/Otu.association).

### Determination of pH, lactic acid, acetic acid, and free amino acids

The pH was measured by using a Sartorius pH indicator. Lactic acid, acetic acid, and free amino acids were determined by using ACQUITY UPLC M-Class System with BEH C18 Column (2.1 × 50 mm × 1.7 μm) and PDA detector (Waters Corporation, Milford, MA, USA). The supernatants of Suanzhou samples were subjected to filtration by the syringe filter (0.2 μm pore size). The filtrate was directly used for the lactic acid and acetic acid analysis. The mobile phase used was prepared by mixing 0.01 mol/l KH_2_PO_4_ (pH 3.0) and CH_3_CN in a ratio of 98:2. Free amino acids in the supernatant samples were determined according to the manual from Waters and the method described previously (Fiechter et al., 2011). The reagent 6-aminoquinolyl-N-hydroxysuccinimidyl carbamate (AQC, Waters) was used to derivatize amino acids. Amino acids AAS18 and A9906 (Sigma-Aldrich) were used as analytical standards. All experiments were repeated three times. The values for pH, organic acids, and free amino acids were subjected to one-way analysis of variance (ANOVA) by Tukey's method of the statistical software Statistica 7.0.

### Isolation and identification of the microorganisms from Suanzhou

Serial dilutions of Suanzhou samples with 0.9% NaCl (normal saline) were prepared. The dilutions were spread on the plates with the selective media, including *Lactobacilli* MRS agar (pH 5.0) for the isolation of LAB (De Man et al., 1960), GYC agar with CaCO_3_ for the isolation of acetic acid bacteria (AAB; Raspor and Goranovic, 2008), and YEPD agar for the isolation of yeast species (Treco and Lundblad, 2001). The plates were incubated at 30°C for 48 h to enumerate the colonies. All experiments were repeated three times. Average and standard deviation (STDEV) were calculated using Excel.

Bacterial genomic DNA was extracted with the E.Z.N.A.® Bacterial DNA Kit (Omega Bio-tek Inc., USA) from a wide variety of gram positive and negative bacterial species. Primers 27f (5′ AGAGTTTGATCC TGGCTCAG 3′) and 1492r (5′ GGTTACCTT GTTACGACTT 3′) were used for amplification of the 16S rRNA gene (Lane, 1991). Fungal genomic DNA was extracted with the method as described previously (Löffler et al., 1997). Primers NS1 (5′ GTAGTCATATGC TTGTCTC 3′) and NS4 (5′ CTTCCGTCA ATTCCTTTAAG 3′) were used to amplify the 18S rRNA gene (White et al., 1990). The amplicons were sequenced directly and the obtained sequences were deposited in GenBank. The accession number of 16S rRNA gene sequences was KX150543 through KX150609 for the LAB isolates and KX150610 through KX150652 for the AAB isolates. The accession number of 18S rRNA gene sequences was KX150653 through KX150704 for the yeast isolates. Sequence similarity was analyzed by using the online tool BLAST in the NCBI (http://blast.ncbi.nlm.nih.gov/Blast.cgi). Sequences were assigned to species level when similarities were at 97% or higher. The phylogenetic tree was constructed with the software MEGA6 (Tamura et al., 2013).

### Growth curve of LAB strains

The growth curve of the isolated LAB isolates was determined in MRS medium (pH 6.0). The single colony was inoculated in 3 ml fresh MRS medium for static cultivation at 30°C for 12 h. The overnight precultures were diluted in fresh MRS medium to OD_600_ <0.2. The mixtures were dispensed into the 96-well plates with 250 μl per well. The culture was then grown for static cultivation at 30°C for 48 h. The OD_600_ was recorded at the 10-min intervals by the Synergy H1 Multi-Mode Reader (BioTek Instruments, Inc., Winooski, VT, USA). All experiments were repeated three times. Average and STDEV were calculated using Excel.

### In-lab fermentation of Suanzhou

Proso millet (100 g) was weighed and cleaned with water. After drying, proso millet was put in a 1-l bottle and filled with 900 ml distilled water. The mixture was pasteurized at 65°C for 30 min and ready for in-lab fermentation of Suanzhou. Two of the isolated LAB strains were selected and used as starter separately. Overnight culture of each strain was transferred in the sterilized proso millet suspension with 5% inoculation for static cultivation at 30°C. The fermented grains were replaced with fresh raw proso millet daily. As aforementioned, Suanzhou samples were analyzed in terms of pH, lactic acid, and free amino acids. LAB cells were enumerated by the standard plating method. The experiment was repeated three times. Average and STDEV were calculated using Excel.

## Results

### Biochemical characteristics of Suanzhou samples

A collection of 59 Suanzhou samples were subjected to the analysis of acidity, lactic acid, and acetic acid. The pH value ranged from 3.22 ± 0.01 to 5.15 ± 0.02 in all samples (Table S1). The lactic acid concentration was from 0.74 ± 0.02 to 6.20 ± 0.04 mg/ml in the samples fermented from proso millet and 2.93 ± 0.00 to 17.00 ± 0.00 mg/ml in the samples from millet (Table S1). The acetic acid concentration was from 0.33 ± 0.00 to 7.66 ± 0.05 mg/ml in the samples fermented from proso millet and 0.28 ± 0.04 to 3.80 ± 0.00 mg/ml in the samples from millet (Table S1). With statistical analysis, the significant differences were observed in the comparison pairs, including groups A and C, A and D, B and C, and B and D, suggesting the raw materials were associated with the organic acid levels in Suanzhou.

### Analysis of free amino acids content

The content of free amino acids was measured, ranging from 81.93 ± 5.01 to 665.47 ± 2.19 μ g/ml in the samples fermented from proso millet and 67.91 ± 0.41 to 1257.30 ± 0.93 μ g/ml in the samples from millet (Table S1). Of the total amino acids, essential amino acids accounted for 13.27 ± 0.29 to 48.02 ± 0.48% in the samples fermented from proso millet and 27.50 ± 0.09 to 51.81 ± 0.02% in the samples from millet (Table S1). With statistical analysis, Suanzhou fermented from the different cereals displayed the significant difference in the content of free amino acids among the comparison pairs, including groups A and C, A and D, B and C, and B and D. However, rice had no effects on the amino acids levels, possibly due to the low ratio (<10%) in the raw material.

### Analysis of the V4 region of 16S rRNA gene

**Figure 1:**
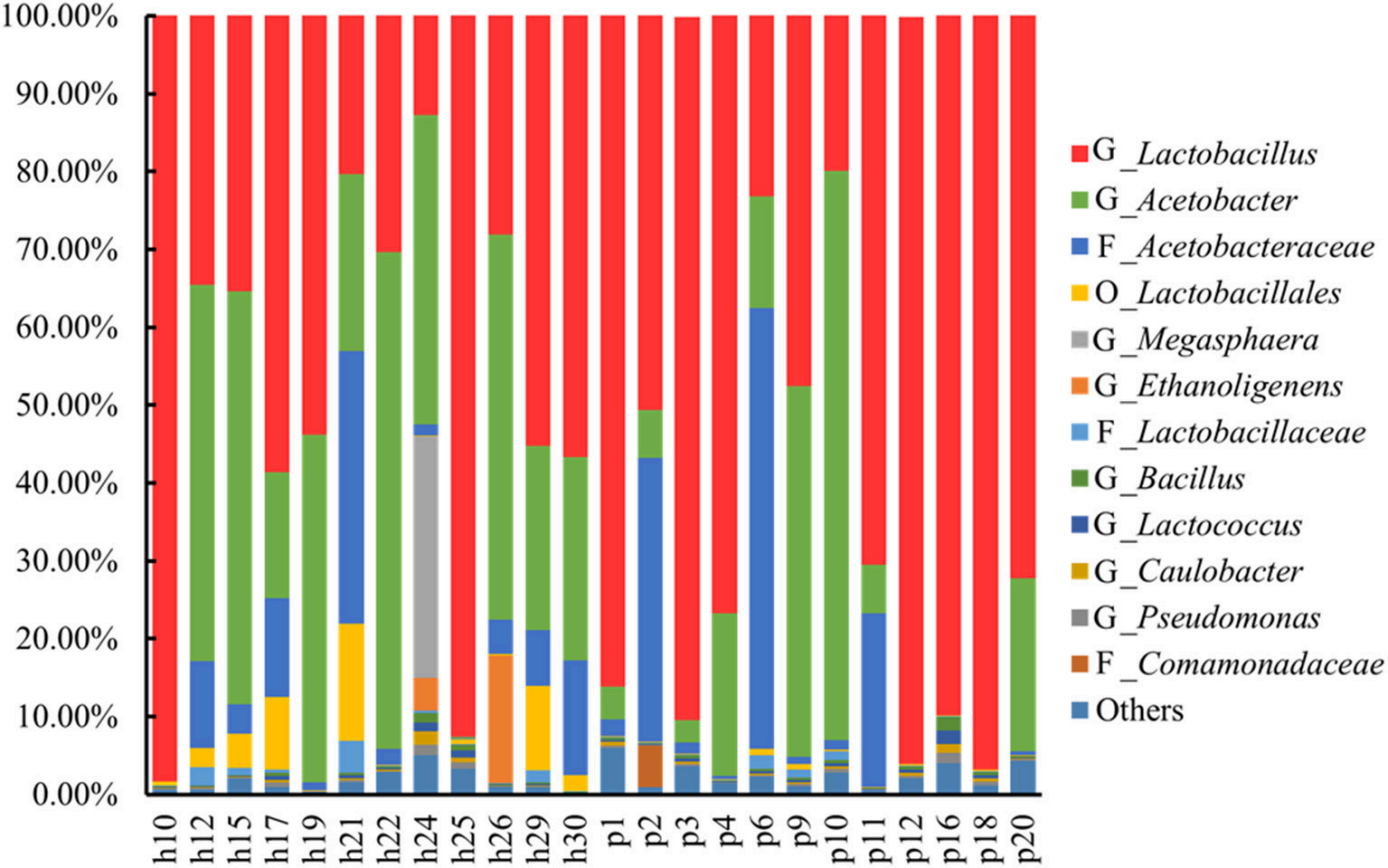
**Abundance of the top 12 abundant operational taxonomic units (OTUs) among the Suanzhou samples**. OTUs with the abundance <0.10% were not included in this diagram. G: OTUs at the genus level. F: OTUs at the family level. O: OTUs at the order level. Others: all OTUs with the abundance ≥0.10% were included. In summary, h10 with 0.10% *Streptophyta* (order); h12 with 0.10% *Psychrobacter* and 0.10% *Clostridium*; h15 with 0.10% *Psychrobacter* and 0.3% *Streptophyta* (order); h17 with 0.30% *Psychrobacter*, 0.10% *Micrococcaceae*, 0.10% *Phenylobacterium*, and 0.10% *Methylobacteriaceae* (family); h21 with 0.20% *Psychrobacter*, 0.10% *Micrococcaceae* (family), 0.10% *Phenylobacterium*, 0.10% *Bacteroides*, and 0.30% Bacteroidales (order); h22 with 0.10% *Psychrobacter*, and 2.60% *Gluconacetobacter*; h24 with 1.30% *Psychrobacter*, 0.20% Micrococcaceae (family); 0.5% *Phenylobacterium*, 0.40% *Methylobacteriaceae* (family), 0.10% *Bacteroides*, 0.10% *Bacteroidales* (order), 0.10% *Brochothrix*, 0.10% *Caulobacteraceae* (family), 0.10% *Balneimonas*, 0.90% *Methylobacterium*, 0.10% *Halomonas*, 0.30% *Acinetobacter*, 0.10% *Enhydrobacter*, and 0.10% *Stenotrophomonas*; h25 with 0.40% *Psychrobacter*, 0.30% *Streptophyta* (order), 0.20% *Phenylobacterium*, 0.10% *Methylobacteriaceae* (family); 0.10% *Bacteroides*, 0.20% *Myroides*, 0.20% *Sphingobacterium*, 0.10% *Bacteroidales* (order), 0.10% *Brochothrix*, 0.20% *Staphylococcus*, 0.10% *Gemellales* (order), 0.10% *Kuenenia*, 0.20% *Enterobacteriaceae* (family), 0.10% *Klebsiella*, 0.20% *Acinetobacter*, 0.10% *Xanthomonadaceae* (family); h26 with 0.10% *Streptophyta* (order); h29 with 0.10% *Psychrobacter*, and 0.50% *Pediococcus*; p1 with 0.40% *Psychrobacter*, 0.10% *Micrococcaceae* (family), 0.40% *Streptophyta* (order), 0.10% *Phenylobacterium*, and 0.10% *Methylobacteriaceae* (family); p2 with 0.10% *Comamonas*, 0.20% *Enterobacteriaceae* (family), 0.10% *Klebsiella*, 0.10% *Moraxellaceae* (family), 0.10% *Acinetobacter*, and 0.10% *Xanthomonadaceae* (family); p3 with 0.20% *Psychrobacter*, 0.10% *Streptophyta*, 0.10% *Phenylobacterium*, 0.10% *Methylobacteriaceae* (family), 0.60% *Bacteroides*, 0.20% *Bacteroidales* (order), 0.30% *Bacteroidia* (class), 0.30% *Barnesiellaceae* (family), 0.10% *Veillonellaceae* (family), 0.10% *Desulfovibri*, and 0.10% *Akkermansia*; p4 with 0.20% *Streptophyta* (order); p6 with 0.20% *Psychrobacter*, 0.10% *Micrococcaceae* (family), 0.10% *Streptophyta* (order), 0.10% *Phenylobacterium*, 0.10% *Methylobacteriaceae* (family), and 0.10% *Bifidobacteriaceae* (family); p9 with 0.20% *Psychrobacter*, 0.10% *Phenylobacterium*, and 0.10% *Methylobacteriaceae* (family); p10 with 0.20% *Psychrobacter*, 0.10% *Micrococcaceae* (family), 0.10% *Phenylobacterium*, 0.10% *Actinomyces*, 1.10% *Chryseobacterium*, 0.20% *Klebsiella*, and 0.30% *Acinetobacter*; p12 with 0.20% *Psychrobacter*, 0.10% *Micrococcaceae* (family), 0.10% *Streptophyta* (order), 0.10% *Phenylobacterium*, 0.20% *Methylobacteriaceae* (family), 0.10% *Bacteroidales* (order); p16 with 0.80% *Psychrobacter*, 0.30% *Micrococcaceae* (family), 0.30% *Streptophyta* (order), 0.30% *Phenylobacterium*, 0.30% *Methylobacteriaceae* (family), 0.10% *Nocardioidaceae* (family), 0.10% *Myroides*, 0.10% *Brochothrix*, 0.10% *Paenibacillus*; p18 with 0.30% *Psychrobacter*, 0.10% *Micrococcaceae* (family), 0.10% *Phenylobacterium*, 0.10% *Methylobacteriaceae* (family), 0.10% *Halomonas*; and p20 with 0.10% *Psychrobacter*, 0.10% *Streptophyta* (order), 0.40% *Halomonas*, and 0.20% *Thermus.*

The species diversity, richness, and evenness in the 24 Suanzhou samples were estimated by the collector rarefaction curves of the observed species, chao1, and Shannon indices (Figure S1). The results showed that the libraries were relatively well-sampled and constructed. The bacterial diversity was mainly analyzed at the genus level. In the 24 Suanzhou samples, the top 4 dominant species groups were the genus *Lactobacillus*, the genus *Acetobacter*, the family *Acetobacteraceae*, and the order *Lactobacillales*, with an average abundance of 58.20 ± 0.28, 24.40 ± 0.23, 9.00 ± 0.15, and 2.00 ± 0.04%, respectively (Figure 1). The sample h25 had 55 OTUs, the maximum of all tested samples; while only 21 OTUs were found in the sample h19, mainly including *Lactobacillus* (53.80%) and *Acetobacter* (44.7%) (Figure S2).

There were many microorganisms commonly present in the Suanzhou samples with low abundance. *Psychrobacter* species were detected in 17 Suanzhou samples with the average abundance from 0.10 to 1.30%. *Phenylobacterium* species were detected in 12 Suanzhou samples with the average abundance from 0.10 to 0.50%. *Streptophyta* (order) species were detected in 11 Suanzhou samples with the average abundance from 0.10 to 0.40%. *Methylobacteriaceae* (family) species were detected in 11 Suanzhou samples with the average abundance from 0.10 to 0.40%. *Micrococcaceae* (family) species were detected in 9 Suanzhou samples with the average abundance from 0.10 to 0.30% (Figure 1).

### Analysis of the ITS1 region

**Figure 2:**
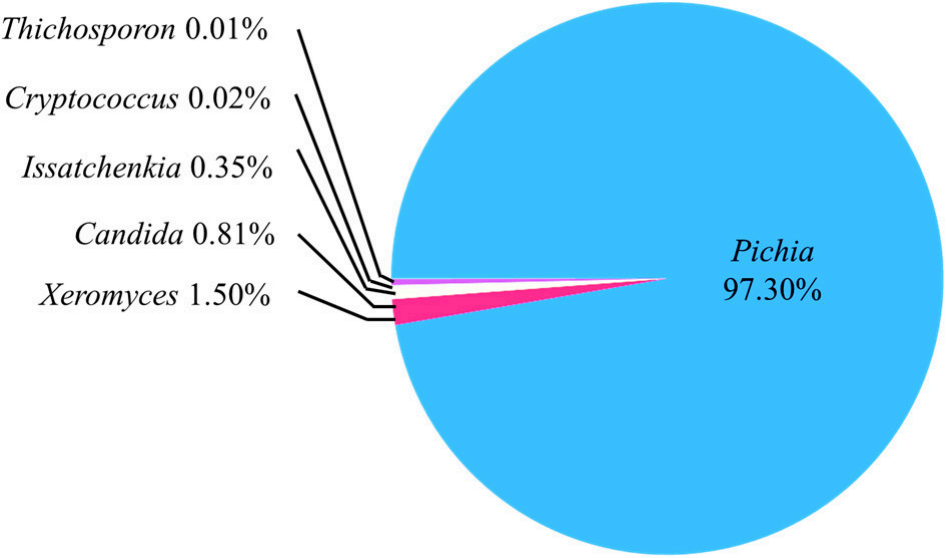
**Abundance of the operational taxonomic units (OTUs) on the basis of IST1 region analysis**. All OTUs were at the genus level. The sequences with no blast hits (29.43% of the total clean reads) weren't included in this pie-chart.

The sample h21 was analyzed for its fungal communities by the Miseq system. In the obtained sequences, 29.40% showed no blast hits. Of the remaining sequences, at the genus level, the abundance of *Pichia, Xeromyces, Candida, Issatchenkia, Cryptococcus*, and *Trichosporon* accounted for 97.30, 1.50, 0.81, 0.35, 0.02, and 0.01%, respectively (Figure 2). *Pichia* was the prominent OTUs in the sample h21.

In contrast to the fungal population analysis, the bacterial diversity was also determined in the sample h21. The dominant OTUs included *Acetobacteraceae* (family) 35.0%, *Lactobacillales* (order) 15.0%, *Acetobacter* 22.8%, *Lactobacillus* 20.3%, and *Lactobacillaceae* (family) 4.1% (Figure 1).

### Enumeration and identification of LAB isolates

**Figure 3:**
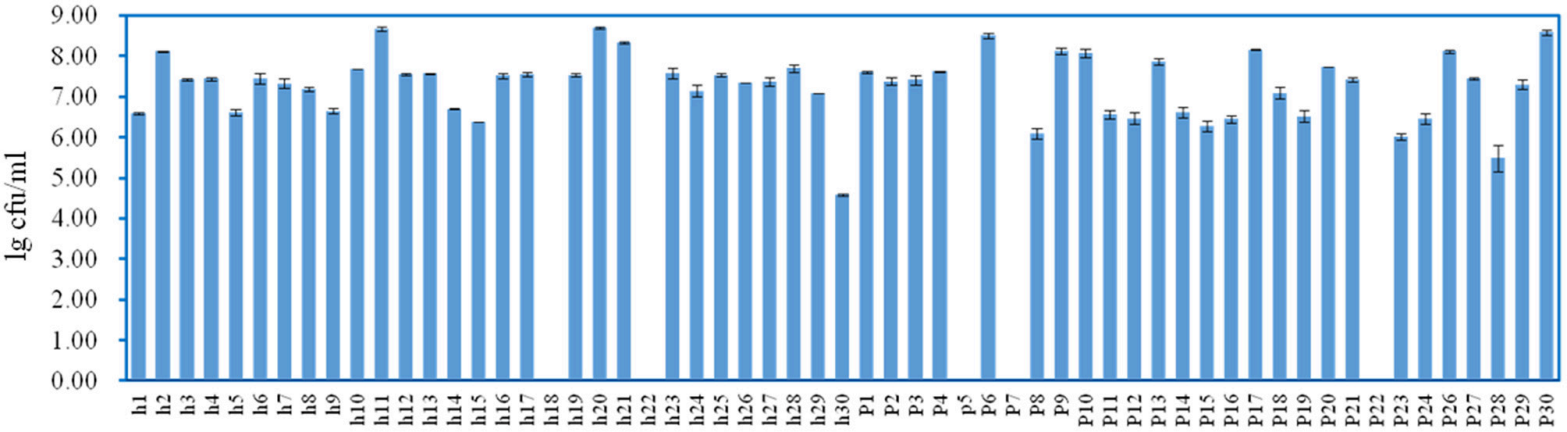
**Enumeration of lactic acid bacteria (LAB) in the Suanzhou samples**.

**Figure 4:**
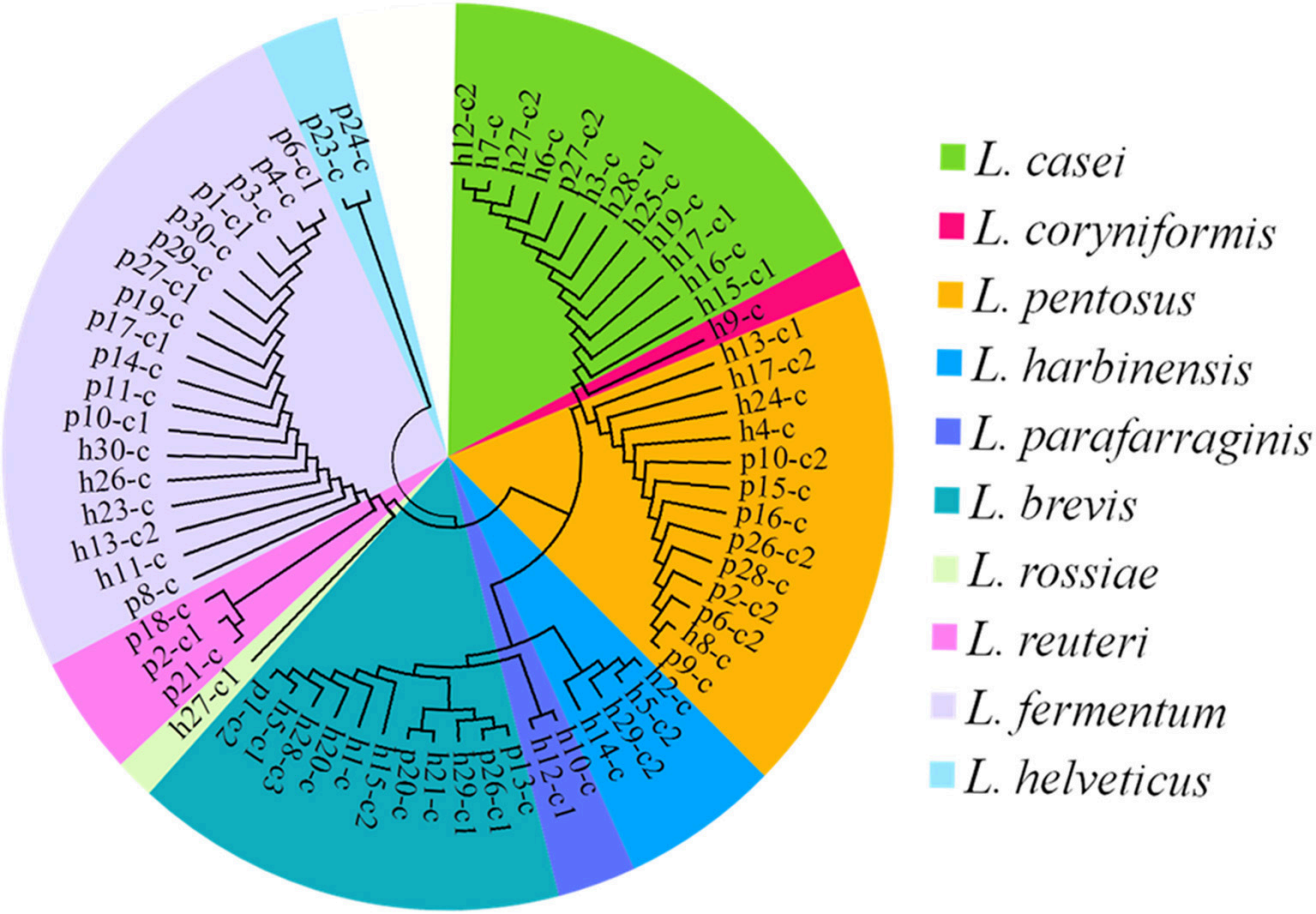
**The cladogram tree of the lactic acid bacteria (LAB)**. The partial 16S rRNA gene sequences (921–925 bp) were analyzed using the Neighbor-Joining method. L, *Lactobacillus*.

White transparent or opaque colonies on the MRS agar plates were counted. Totally 67 presumptive LAB strains were isolated and the corresponding bacteria concentrations ranged from 4.58 ± 0.03 lg cfu/ml (sample h30) to 8.69 ± 0.05 lg cfu/ml (sample h20) except that no colonies were observed with the samples h18, h22, p5, p7, and p22 (Figure 3). The 16S rRNA gene sequence of the LAB isolates was analyzed. All isolates were identified as the members of the genus *Lactobacillus*, including *L. brevis* (11), *L. casei* (12), *L. coryniformis* (1), *L. fermentum* (18), *L. harbinensis* (4), *L. helveticus* (2), *L. parafarraginis* (2), *L. pentosus* (13), *L. reuteri* (3), and *L. rossiae* (1). Fourteen samples had two *Lactobacillus* species coexisted in the same gruel fermentor (Figure 4, Table 1).

**Table 1:** **Phylogenetic affiliations of the isolates**.

**No**.	**Closest sequence of LAB**	**Closest sequence of AAB**	**Closest sequence of yeast**
h27	*L. rossiae, L. casei*	*A. lovaniensis*	*P. membranifaciens*
p2	*L. reuteri, L. pentosus*	*A. papayae*	*G. geotrichum*
p18	*L. reuteri*		*P. kudriavzevii*
p21	*L. reuteri*		
h13	*L. pentosus, L. fermentum*	*A. okinawensis*	*P. kudriavzevii*
p15	*L. pentosus*		*P. kudriavzevii*
h4	*L. pentosus*	*A. lovaniensis*	*P. kudriavzevii, G. geotrichum*
h8	*L. pentosus*	*A. lovaniensis*	*P. kudriavzevii*
h24	*L. pentosus*	*A. lovaniensis*	*P. kudriavzevii*
p9	*L. pentosus*	*A. lovaniensis*	
p16	*L. pentosus*		*P. kudriavzevii*
p28	*L. pentosus*		*G. geotrichum*
h12	*L. parafarraginis, L. casei*	*A. syzygii*	*P. kudriavzevii*
h10	*L. parafarraginis*		*P. kudriavzevii*
p24	*L. helveticus*	*A. lovaniensis*	*P. kudriavzevii*
p23	*L. helveticus*	*A. lovaniensis*	
h14	*L. harbinensis*	*A. fabarum*	
h2	*L. harbinensis*		*P. kudriavzevii*
p10	*L. fermentum, L. pentosus*	*A. orientalis*	*G. geotrichum*
p6	*L. fermentum, L. pentosus*	*A. malorum*	
p27	*L. fermentum, L. casei*		*P. kudriavzevii*
p1	*L. fermentum, L. brevis*		*S. cerevisiae*
p17	*L. fermentum*		
p4	*L. fermentum*	*A. lovaniensis*	*P. kudriavzevii*
p14	*L. fermentum*		*P. kudriavzevii*
p11	*L. fermentum*	*A. okinawensis, A. papayae*	
h11	*L. fermentum*	*A. okinawensis*	*G. geotrichum*
h23	*L. fermentum*	*A. lovaniensis, A. okinawensis*	*P. kudriavzevii, G. geotrichum*
h26	*L. fermentum*	*A. lovaniensis*	*P. kudriavzevii*
h30	*L. fermentum*	*A. lovaniensis*	*P. kudriavzevii*
p30	*L. fermentum*	*A. indonesiensis*	*P. kudriavzevii, G. geotrichum*
p3	*L. fermentum*	*A. indonesiensis*	*P. kudriavzevii*
p19	*L. fermentum*		*G. geotrichum*
p29	*L. fermentum*		*G. geotrichum*
p8	*L. fermentum*		
h9	*L. coryniformis*	*A. lovaniensis*	*P. kudriavzevii*
h17	*L. casei, L. pentosus*	*A. indonesiensis*	*P. fermentans*,
h15	*L. casei, L. brevis*	*A. okinawensis*	
h28	*L. casei, L. brevis*		*P. membranifaciens*
h6	*L. casei*	*A. syzygii*	*P. occidentalis*
h19	*L. casei*	*A. syzygii*	*P. kudriavzevii*
h25	*L. casei*	*A. senegalensis*	*P. kudriavzevii, P. membranifaciens*
h7	*L. casei*	*A. okinawensis*	*P. kudriavzevii, G. geotrichum*
h16	*L. casei*	*A. lovaniensis*	*P. membranifaciens*
h3	*L. casei*	*A. lovaniensis*	
p26	*L. brevis, L. pentosus*	*A. indonesiensis*	*P. kudriavzevii*
h5	*L. brevis, L. harbinensis*	*A. lovaniensis*	*P. occidentalis*
h29	*L. brevis, L. harbinensis*		*P. fermentans*
h21	*L. brevis*	*A. syzygii*	*P. membranifaciens*
p13	*L. brevis*	*A. okinawensis*	
p20	*L. brevis*	*A. cibinongensis*	*P. kudriavzevii, G. geotrichum*
h20	*L. brevis*		*G. geotrichum*
h1	*L. brevis*		
h22		*A. syzygii, A. lovaniensis*	*P. kudriavzevii*
p22		*A. lovaniensis*	*P. kudriavzevii*
p5		*A. syzygii*	*P. kudriavzevii*
p7		*A. indonesiensis*	
h18			*P. occidentalis*
p12			*P. kudriavzevii*

All isolates display 98–100% identity to the closest sequences. The isolates were arranged according to alphabetical order of LAB species names. L, Lactobacillus; A, Acetobacter; G, Galactomyces; P, Pichia; S, Saccharomyces; LAB, lactic acid bacteria; AAB, acetic acid bacteria. Gluconobacter oxydans was isolated in the sample h18. Pseudomonas psychrophila and Sphingobacterium mizutaii were isolated in the sample h28. Microbacterium schleiferi was isolated in the sample p14.

### Enumeration and identification of AAB isolates

**Figure 5:**
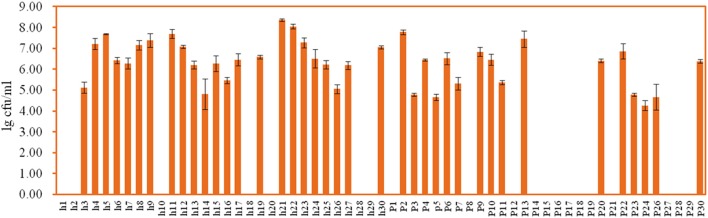
**Enumeration of acetic acid bacteria (AAB) in the Suanzhou samples**.

**Figure 6:**
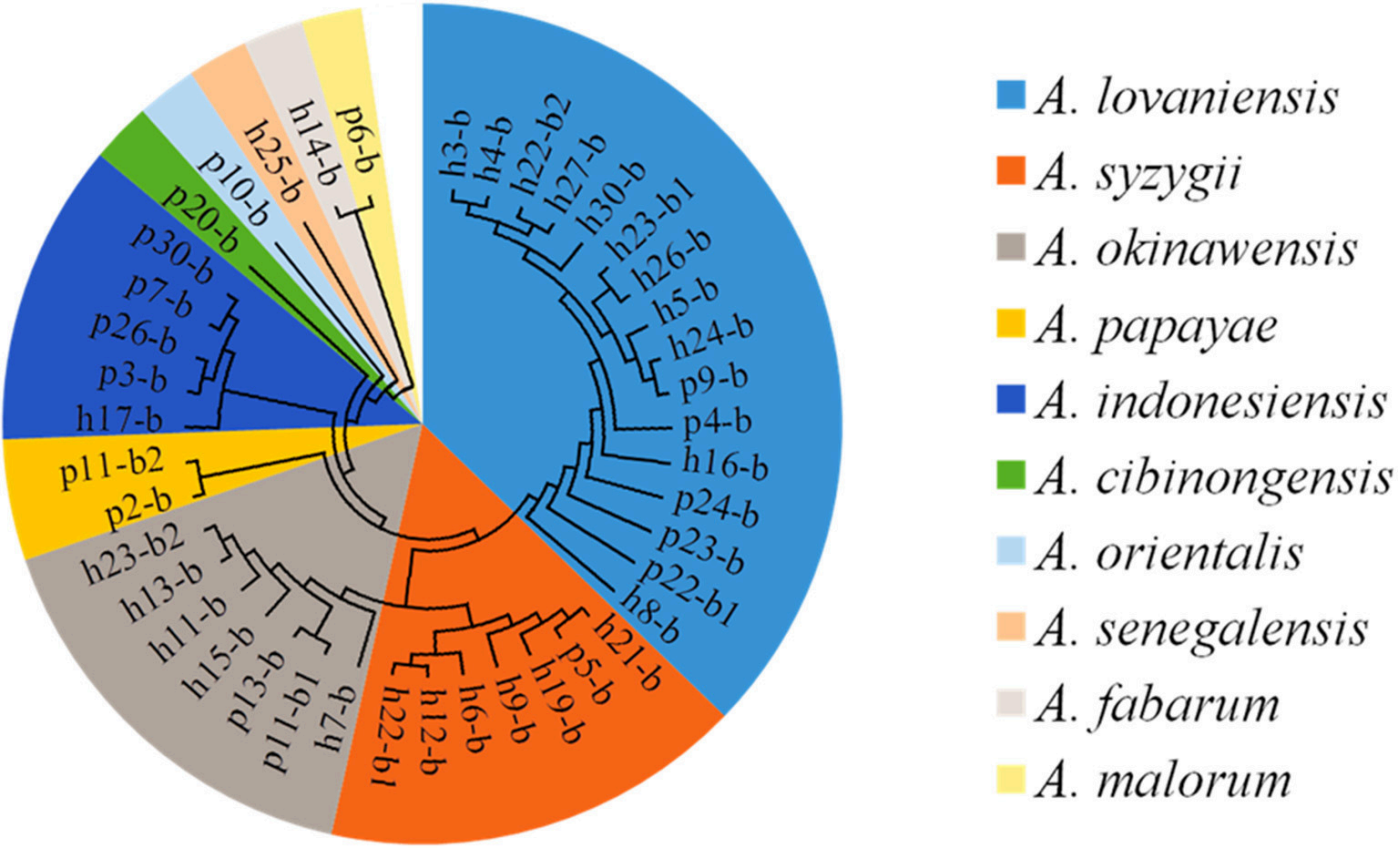
**The cladogram tree of the acetic acid bacteria (AAB)**. The partial 16S rRNA gene sequences (1005–1026 bp) were analyzed using the Neighbor-Joining method. A, *Acetobacter*.

Yellow transparent colonies surrounded with clear zones were counted on the GYC-agar plates. Forty-two isolates were recovered from Suanzhou samples with the concentrations ranging from 4.26 ± 0.24 lg cfu/ml (sample p24) to 8.41 ± 0.17 lg cfu/ml (sample h28) (Figure 5). The 16S rRNA gene sequence of the AAB isolates was analyzed. All isolates were identified as the members of the genus *Acetobacter*, including *A. cibinongensis* (1), *A. indonesiensis* (5), *A. lovaniensis* (16), *A. okinawensis* (7), *A. orientalis* (1), *A. papaya* (2), *A. senegalensis* (1), *A. syzygii* (7), *A. malorum* (1), and *A. fabarum* (1) (Figure 6). *Acetobacter* species were absent in the remaining 20 Suanzhou samples (Table 1). Twenty Suanzhou samples were found absent of *Acetobacter* species. Seventeen of them had no colonies on the GYC plates and the remaining three samples recovered the bacteria other than *Acetobacter* spp., including *Gluconobacter oxydans* in the sample h18, *Pseudomonas psychrophila* and *Sphingobacterium mizutaii* in the sample h28, and *Microbacterium schleiferi* in the sample p14.

### Enumeration and identification of yeast isolates

**Figure 7:**
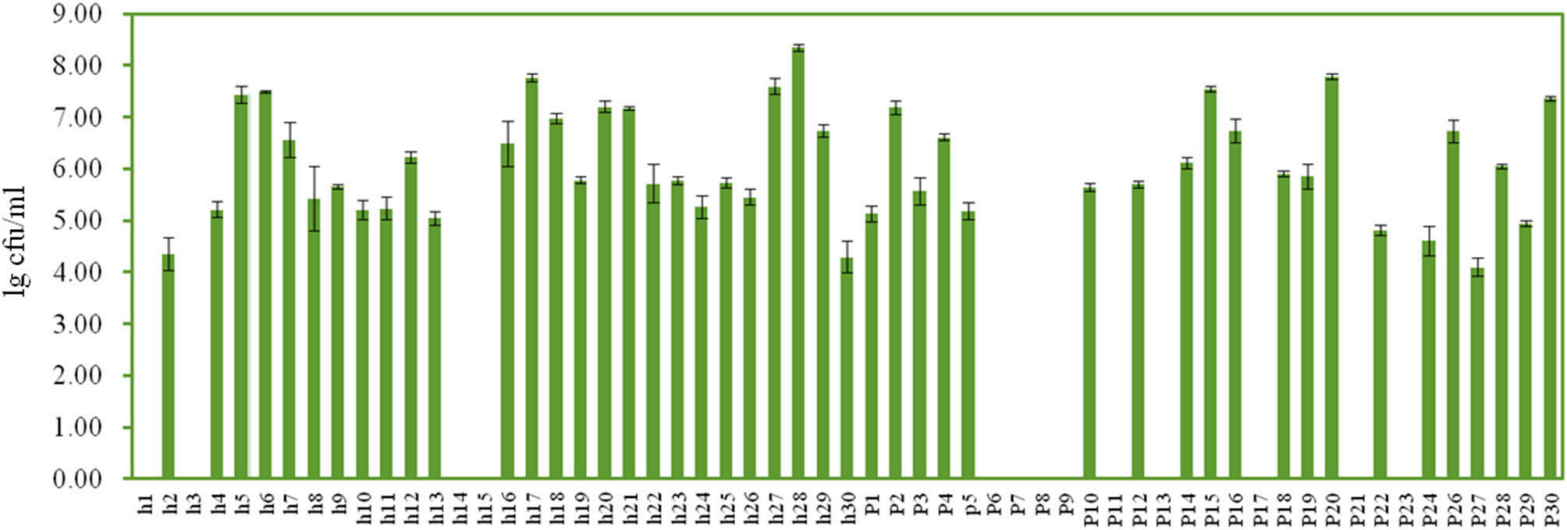
**Enumeration of yeasts in the Suanzhou samples**.

**Figure 8:**
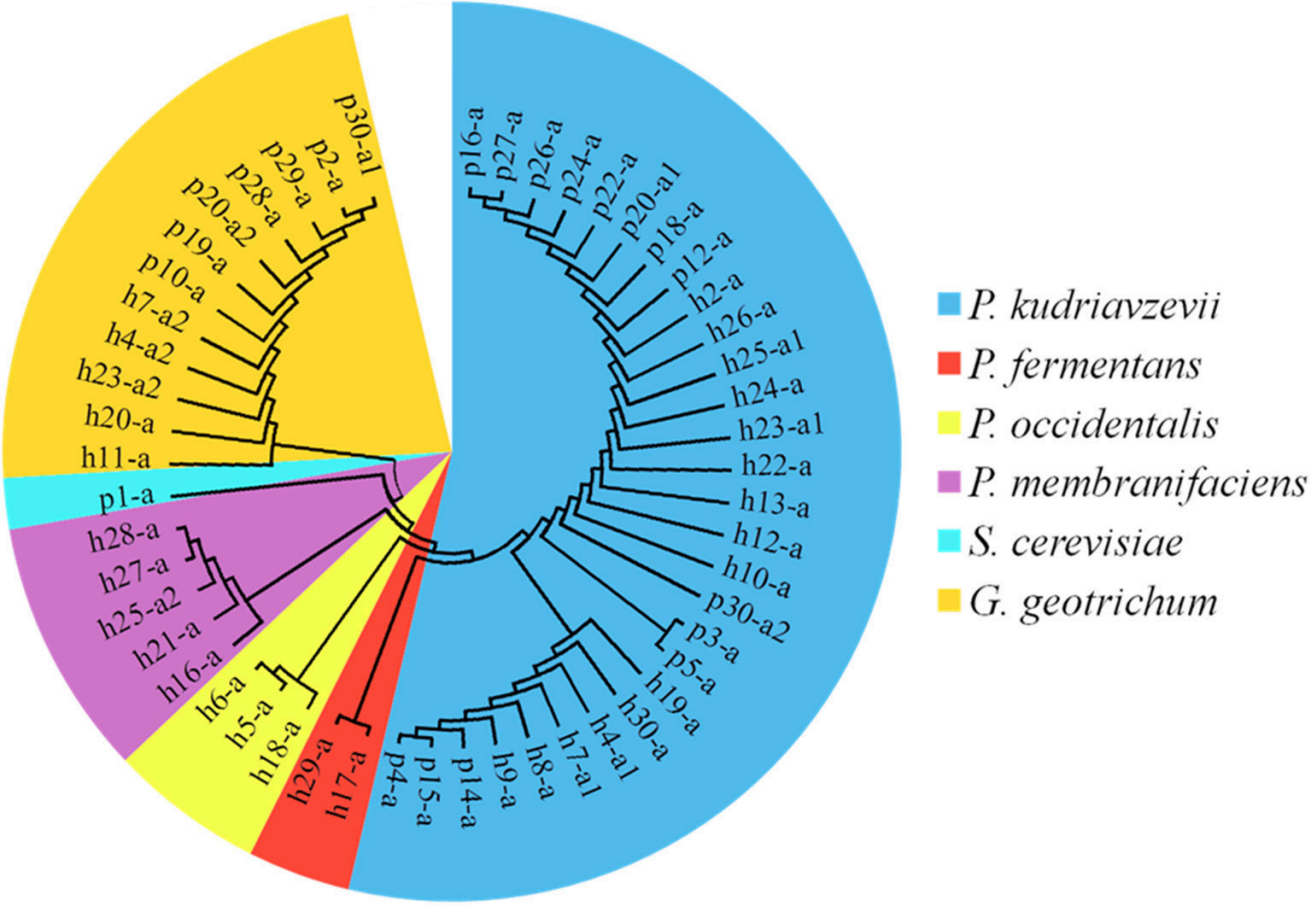
**The cladogram tree of the yeasts**. The partial 18S rRNA gene sequences (928–944 bp) were analyzed using the Neighbor-Joining method. P, *Pichia*. S, *Saccharomyces*. G, *Galactomyces*.

On the YEPD-agar plates, three types of colonies were counted, large yellow colonies, large white colonies, and small white colonies with hyphae. The concentrations of the isolates ranged from 4.10 ± 0.17 lg cfu/ml (sample p27) to 8.35 ± 0.07 lg cfu/ml (sample h28), and no isolates were found in 13 Suanzhou samples (Figure 7). The 18S rRNA gene sequence of the yeast isolates was analyzed. All isolates were classified into 3 genera, including *Pichia fermentans* (2), *P. kudriavzevii* (29), *P. membranifaciens* (5), *P. occidentalis* (3), *S. cerevisiae* (1), and *Galactomyces geotrichum* (12) (Figure 8, Table 1).

### Growth curves of the LAB strains

*L. fermentum, L. pentosus, L. casei*, and *L. brevis* were the most frequently isolated strains from Suanzhou and 31 of them were selected for the growth study (Table 1). As inferred from the growth curves, all strains were divided into three groups based on their growth rates. Group I strains had the lag phase shorter than 1 h and their highest cell density was at 1.3–1.6 of OD_600_, including 10 *L. pentosus* strains and *L. casei* h6-c, h16-c, h17-c1, and h28-c1. Group II strains had the lag phase of 2–3 h and their highest cell density was at 1.2–1.4 of OD_600_, including 10 *L. brevis* strains and *L. casei* h3-c, h25-c, h27-c2, and p27-c2. Group III strains grew very slowly without perceptible lag phases and their highest cell density was at 0.2–0.4 of OD_600_, including *L. fermentum* p17-c1, *L. fermentum* p27-c1, and *L. casei* h12-c2 (Figure S3).

### In-lab fermentation of Suanzhou

**Figure 9:**
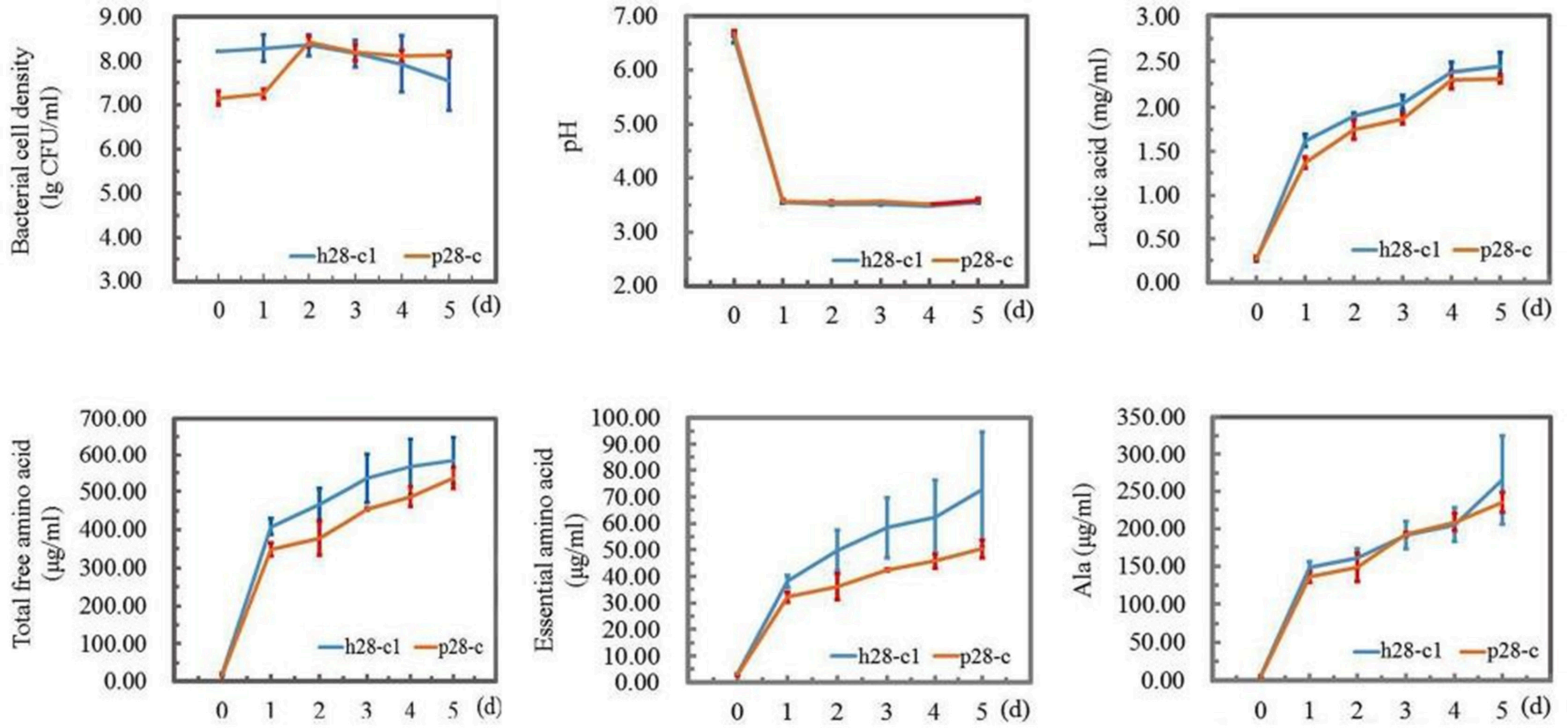
**Preparation of Suanzhou with in-lab fermentations**. Strain *L. casei* h28-c1 and *L. pentosus* p28-c were used as starters. Bacterial cell density (lg cfu/ml), pH, lactic acid, total free amino acid, essential amino acid, and alanine content were evaluated, respectively.

Two fast growing strains *L. pentosus* p28-c and *L. casei* h28-c1 were selected for a 5 d-fermentation mimicking the natural process for Suanzhou preparation. Proso millet was used as raw material and the relevant parameters were analyzed daily. With 5% innoculum, the population of *L. casei* h28-c1 decreased one log unit after 5 days of fermentation, while the population of *L. pentosus* p28-c increased from 7.16 ± 0.16 to 8.44 ± 0.11 lg cfu/ml after 2 d cultivation (Figure 9). The pH value of the two cultures decreased from about 6.6 to 3.5 after 1-day fermentation and remained constant, consistent with the elevated lactic acid content in the cultures (Figure 9). The amount of the total amino acids, essential amino acids, and alanine increased significantly during the fermentation. Strain *L. casei* h28-c1 produced higher amount of free amino acids and essential amino acids than strain *L. pentosus* p28-c (Figure 9).

## Discussion

**Figure 10:**
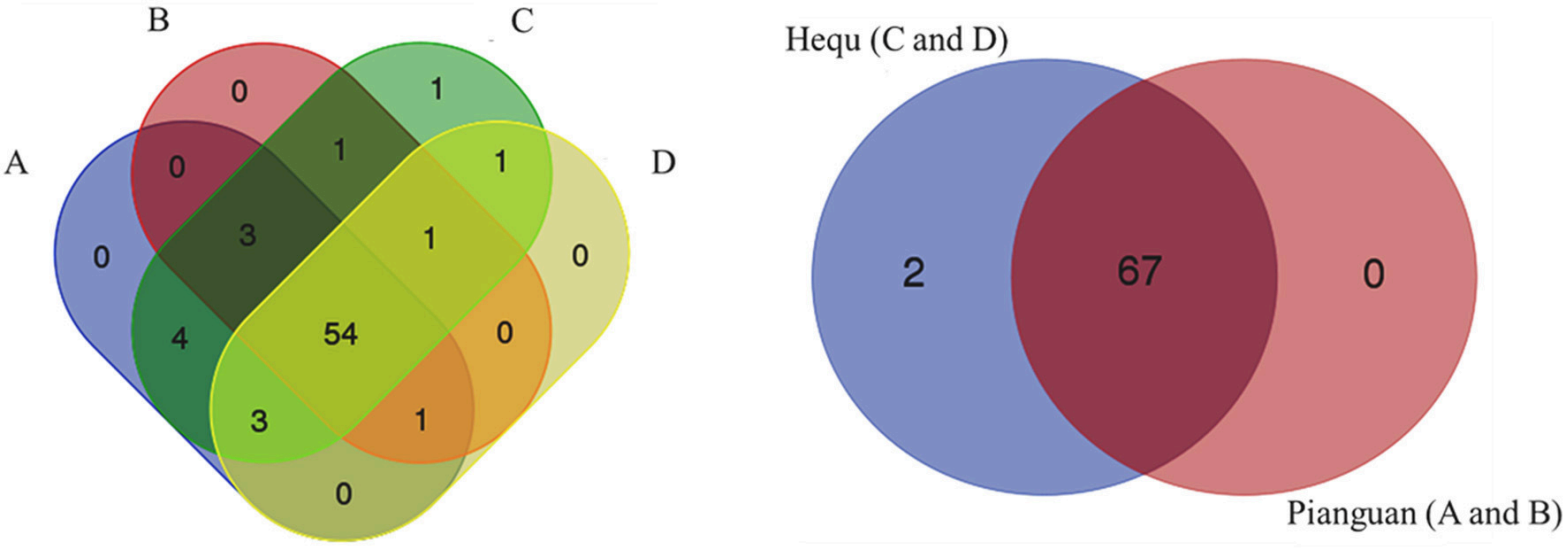
**Venn diagram analysis of the logic relationships of the OTUs identified in the different samples**. The left panel represented the OTUs from the samples fermented with different cereal grains, A: millet and a small amount of rice; B: millet; C: white proso millet; and D: red proso millet. The right panel represented the OTUs from the samples from two different counties: group A and B from Pianguan county, and group C and D from Hequ county.

**Figure 11:**
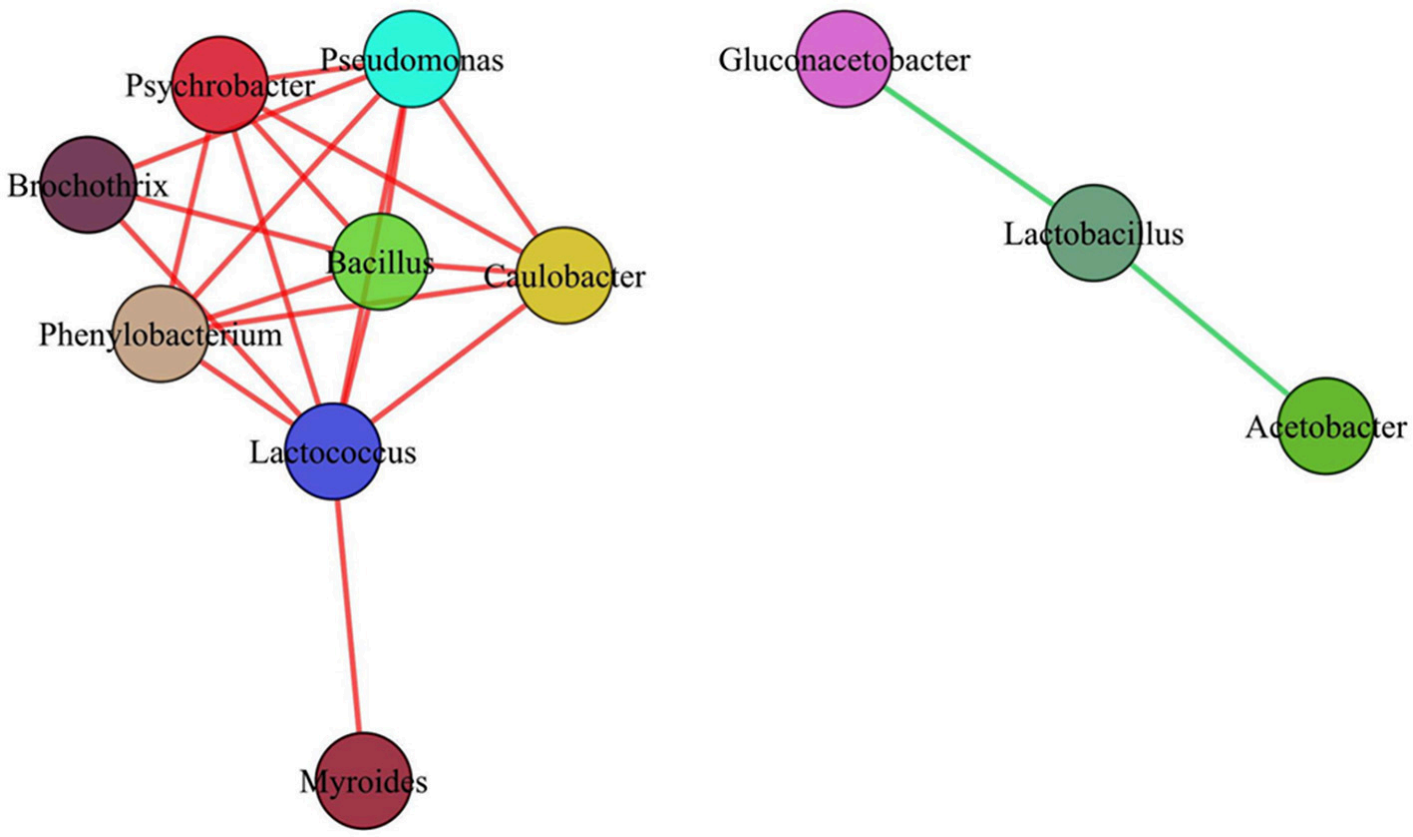
**Implications of the antagonistic or synergistic interactions between the microbial populations**. Green lines represented the antagonistic relationship between the two microbial populations. Red lines represented the synergistic relationship between the two microbial populations.

In our work, totally 69 OTUs at the genus level were detected in Suanzhou samples with the metagenomic analysis. The possible logic relationship of the OTUs identified among the samples was analyzed with the Venn diagram (Figure 10). The samples were divided into group A, B, C, and D based on the cereals, which were made of millet with a small amount of rice, millet, white proso millet, and red proso millet, respectively (Table S1). Fifty-four of them were found in all groups and 67 OTUs found in the samples from both counties, indicating no significant difference existed. The associations among the microbial populations in the samples were predicted. Of the top 20 abundant OTUs found in the metagenomic analysis, the genera *Lactobacillus, Acetobacter*, and *Gluconacetobacter* had no correlations with the remaining 17 OTUs. However, the abundance of *Lactobacillus* was inversely correlated with *Acetobacter* and *Gluconacetobacter*, respectively (Figure 11). Could the antagonistic effect between the genera *Lactobacillus, Acetobacter*, and *Gluconacetobacter* lead to the dying off of any strains was still a question remained be answer by in-lab fermentations using the isolated microbes.

The majority of environmental microorganisms are inculturable with the available methods. In Suanzhou samples, only a few species including LAB, AAB, and yeasts were found with the culture-dependent methods, much less than the OTUs identified with the metagenomic analysis. Furthermore, with the metagenomic analysis, microbial structure was found not unique among Suanzhou samples and some special OTUs were detected in the individual samples (Figure 1). *Gluconacetobacter* was detected in the sample h22 with the abundance of 2.6%, which was commonly the dominant bacterium found in the traditional vinegar production (Hommel, 2014). *Akkermansia* was detected in the sample p3 with a low abundance 0.10%. *A. muciniphila* is the type species of this genus and related with the diet-induced obesity (Everard et al., 2013). *Megasphaera* species was dominant in the sample h24 with an abundance of 31.00%. It's a strictly anaerobic microorganism and the members of this genus were usually recovered from the gastrointestinal tracts of animals (Stanton and Humphrey, 2011; Shetty et al., 2013). *Megasphaera paucivorans* sp. nov. and *Megasphaera sueciensis* sp. nov. were the two new species isolated from brewery samples, probably causing beer spoilage (Juvonen and Suihko, 2006). *Ethanoligenens* was present in two samples h24 and h26 with an abundance of 4.2 and 16.4%, respectively (Figure 1). *Ethanoligenens harbinense* was the only known species relevant to the biomass conversion and biofuels production (Hemme et al., 2010; Liu et al., 2015). The unexpected microorganisms were also found in cultivation process in our work, such as *G. oxydans* (De Muynck et al., 2007), *P. psychrophila* (Abraham and Thomas, 2015), *S. mizutaii* (Wauters et al., 2012), and *M. schleiferi* (Gneiding et al., 2008). These microorganisms were coexisted in Suanzhou at relatively high abundance and some of them may pose potential risks to food safety and human health. It's an innate disadvantage for household-scale fermentation and can be overcome by industrial production using well-characterized starter strains with good manufacturing practice (GMP).

All Suanzhou samples were collected from the household fermentors which had been operating for at least 2 months. In combined with the culture-dependent methods and sequence analyses, totally 10 *Lactobacillus* species, 10 *Acetobacter* species, 1 *G. oxydans* strain, 4 *Pichia* species, 12 *G. geotrichum* strains, and 1 *S. cerevisiae* strain were found in Suanzhou. The results are consistent with the previous findings in a variety of the fermented foods and beverages (Goerges et al., 2008; Eida et al., 2013). Twenty Suanzhou samples had no *Acetobacter* species identified and it may be due to the existence of bacteriocins synthesized by LAB strains, which will inhibit the growth of many bacteria (Heng et al., 2007; Gabrielsen et al., 2014). No LAB were isolated in the 5 Suanzhou samples, 4 of them with the presence of *Acetobacter* spp. and h18 with *G. oxydans* (Table 1), indicating LAB strains may not be the sole organism for Suanzhou production.

Tiny LAB colonies were observed on the MRS plates spread with the sample p12, however, the colonies failed in growth with the subsequent cultivations. The metagenomic analysis showed that p12 was rich in LAB with the total OTUs abundance of 96.20%, including *Lactobacillus, Lactobacillaceae*, and *Lactobacillales* (Figure 1). The growth failure of the pure culture was possibly due to the special nutrient requirements of the LAB isolate, whereas the yeast strain *P. kudriavzevii* in Suanzhou may provide the nutrients essential for the growth of the isolate (Assohoun-Djeni et al., 2016). Strain *L. fermentum* p17-c1, *L. fermentum* p27-c1, and *L. casei* h12-c2 also exhibited low growth rates in the pure cultures, significantly lower than the relevant cell numbers in the corresponding Suanzhou samples (Figure 3 and Figure S3). The results indicated that the Suanzhou micro-ecosystems possibly provided necessary nutrients for the growth of microorganisms either by degradation of the cereals or by the biosynthesis.

In our work, several yeasts were identified. *Pichia* species were commonly isolated in the most samples of Suanzhou without detecting other fungi. The result is consistent with the previous findings that *Pichia* species can antagonize and decrease the abundance of a number of yeast and mold pathogens in various niches (Golubev, 2006; Mukherjee et al., 2014). *G. geotrichum* was coexisted with *Pichia* species in 5 Suanzhou samples, showing that its growth wasn't affected by *Pichia*, also consistent with the previous findings (Viljoen, 2006). *G. geotrichum* or its anamorph *Geotrichum candidum* was widely present in the early stages of ripening on soft cheeses (Marcellino et al., 2001) and some strains were the starter organisms for fermented foods and beverages (Goerges et al., 2008; Tamang et al., 2016a). Only one *S. cerevisiae* strain was isolated from the sample p1 which was absent of *Acetobacter* strains (Table 1). The data is consistent with the fact that acetic acid can suppress the growth of *S. cerevisiae* in sour dough (Suihko and Mäkinen, 1984). Taken together, the yeast strains could play an inhibitory role against food-borne pathogens and also provide some nutrients for the growth of other microbes (Goerges et al., 2006; Viljoen, 2006).

LAB strains have been widely used in fermented food industry as starters (Giraffa et al., 2010). In our wok, 2 *lactobacillus* strains were used for in-lab fermentations separately. The results showed that the in-lab products were similar to traditional gruel in terms of pH value, organic acids, and free amino acids, indicating that *Lactobacillus* species might be one of the key microorganisms for Suanzhou fermentation. In future, co-fermentation with the isolated bacteria and yeast strains will be conducted to select the appropriate candidate microorganisms for possible applications.

Millet is seldom reported as raw material for preparing fermented foods. *Ben-saalga* is a traditional fermented food in Burkina Faso of Africa. It's made from pearl millet by two fermentations, one happens in the step of grains soaking and the other happens in the settlement step of wet flour. Indian *dosa* is another traditional fermented food made from co-fermentation of finger millet and horse gram flour. Both foods are prepared from flour, while Suanzhou is prepared from intact grains. All the three foods are rich in amino acids and can provide diet proteins to infants and young children. With culture-dependent methods, LAB and yeasts were found dominant in both the fermented foods, similar to our findings. However, no molecular identification of the isolates was conducted in both studies.

In conclusion, Suanzhou gruel displayed low acidity and relatively high-level of free amino acids and organic acids. The microbiota in Suanzhou was rich in *Lactobacillus, Acetobacter, Pichia*, and *G. geotrichum* strains. The characteristics of Suanzhou can assure the fermented food to be devoid of many environmental molds, yeasts, and bacteria pathogens and keep the Suanzhou food safely for consumption. The isolated strains may be further characterized and used as starters in the industrial production of Suanzhou food and other applications.

## Author contributions

HQ carried out the bioinformatic analysis and the experiments and wrote the manuscript. QS and XP performed the bioinformatic analysis. ZQ designed the experiments. HY designed the experiments and wrote the manuscript.

### Conflict of interest statement

The authors declare that the research was conducted in the absence of any commercial or financial relationships that could be construed as a potential conflict of interest.

## Supplementary material

Table S1 **Biochemical analysis of the acid-gruel samples**.

Figure S1 **The collector retraction curves of the observed species, Chao1, and Shannon indice of the metagenomic libraries of the 24 Suanzhou samples**.

Figure S2 **The OTUs distributions in the 24 Suanzhou samples**.

Figure S3 **Growth curves of the isolated lactic acid bacteria**.
