# Supplementary material for: Microbial Diversity and Biochemical Analysis of Suanzhou: A Traditional Chinese Fermented Cereal Gruel
Source: Front Microbiol. 2016 Aug 25;7:1311. doi: 10.3389/fmicb.2016.01311 (PMC4997791; doi:10.3389/fmicb.2016.01311)
Supplement: Table S1 — Biochemical analysis of the acid-gruel samples. [file Table1.doc]

Table S1 Biochemical analysis of the acid-gruel samples

| Samples | Cereals | pH | Lactic acid | Acetic acid | Free aa | Relative amount | Relative amount |
| --- | --- | --- | --- | --- | --- | --- | --- |
|  |  |  | (mg/ml) | (mg/ml) | (μg/ml) | of Ala (%) | of essential aa (%) |
| h1 | RPM | 3.59±0.01 | 5.91±0.02 | 1.33±0.00 | 307.43±1.33 | 19.20±0.08 | 23.41±0.01 |
| h2 | WPM | 3.22±0.01 | 6.01±0.02 | 2.99±0.02 | 124.21±1.28 | 9.78±0.07 | 36.49±0.04 |
| h3 | WPM | 3.54±0.01 | 4.91±0.02 | 5.91±0.02 | 415.40±3.41 | 33.49±0.42 | 22.19±0.13 |
| h4 | WPM | 3.92±0.01 | 1.66±0.02 | 2.25±0.00 | 207.77±4.86 | 42.68±0.06 | 23.10±0.19 |
| h5 | WPM | 4.82±0.01 | 1.18±0.00 | 0.33±0.00 | 317.96±9.05 | 21.30±0.25 | 48.02±0.48 |
| h6 | WPM | 3.82±0.01 | 3.46±0.02 | 3.59±0.02 | 394.01±4.92 | 36.48±0.02 | 25.94±0.32 |
| h7 | RPM | 4.33±0.02 | 1.71±0.02 | 2.36±0.02 | 467.55±6.01 | 28.30±0.10 | 37.02±0.43 |
| h8 | WPM | 3.96±0.01 | 1.88±0.00 | 2.31±0.02 | 210.15±2.57 | 33.59±0.06 | 27.86±0.28 |
| h9 | WPM | 4.02±0.01 | 1.93±0.00 | 2.76±0.02 | 236.02±2.81 | 32.64±0.09 | 32.08±0.32 |
| h10 | WPM | 3.64±0.01 | 5.91±0.02 | 2.64±0.02 | 239.89±1.92 | 31.80±0.21 | 13.27±0.29 |
| h11 | WPM | 4.57±0.00 | 1.24±0.02 | 0.55±0.00 | 244.18±2.39 | 26.00±0.24 | 42.98±0.08 |
| h12 | WPM | 4.13±0.01 | 0.74±0.02 | 2.00±0.00 | 141.92±4.39 | 44.27±0.41 | 27.15±0.29 |
| h13 | WPM | 3.46±0.02 | 3.88±0.00 | 0.98±0.00 | 81.93±5.01 | 48.07±0.18 | 13.83±0.31 |
| h14 | WPM | 3.98±0.01 | 2.14±0.02 | 7.66±0.05 | 578.69±1.39 | 47.75±0.16 | 31.42±0.09 |
| h15 | RPM | 3.45±0.02 | 6.20±0.04 | 3.40±0.07 | 285.20±5.21 | 52.85±0.04 | 15.69±0.18 |
| h16 | WPM | 3.71±0.01 | 2.36±0.02 | 3.79±0.02 | 168.36±5.16 | 53.51±0.09 | 13.93±0.14 |
| h17 | RPM | 4.35±0.02 | 1.10±0.00 | 2.38±0.04 | 258.82±2.29 | 35.93±0.05 | 38.23±0.31 |
| h18 | RPM | 3.35±0.01 | 4.44±0.02 | 1.15±0.04 | 91.27±1.24 | 32.76±0.26 | 18.42±0.18 |
| h19 | RPM | 3.85±0.00 | 2.93±0.00 | 2.10±0.04 | 202.44±9.01 | 26.41±0.07 | 43.25±0.41 |
| h20 | WPM | 4.09±0.01 | 1.89±0.02 | 1.54±0.02 | 120.23±1.69 | 15.92±0.13 | 39.75±0.07 |
| h21 | RPM | 4.38±0.01 | 0.98±0.00 | 1.26±0.02 | 117.50±4.81 | 28.69±0.25 | 32.19±0.19 |
| h22 | WPM | 4.02±0.01 | 1.75±0.00 | 1.96±0.12 | 230.24±7.82 | 31.02±0.36 | 25.97±0.42 |
| h23 | WPM | 3.83±0.01 | 2.76±0.02 | 3.59±0.02 | 338.54±4.91 | 48.97±0.28 | 16.46±0.29 |
| h24 | WPM | 5.15±0.02 | 2.19±0.02 | 1.38±0.04 | 665.47±2.19 | 47.88±0.61 | 22.13±0.19 |
| h25 | WPM | 3.52±0.00 | 6.09±0.02 | 2.25±0.00 | 293.12±7.99 | 24.51±0.73 | 13.39±0.13 |
| h26 | WPM | 4.36±0.01 | 1.41±0.02 | 2.31±0.02 | 373.48±1.97 | 38.80±0.18 | 30.47±0.08 |
| h27 | WPM | 3.86±0.00 | 2.69±0.02 | 4.00±0.00 | 392.10±4.31 | 53.36±0.27 | 24.16±0.38 |
| h28 | RPM | 4.16±0.01 | 1.31±0.02 | 1.23±0.00 | 159.17±9.21 | 22.59±0.09 | 43.64±0.14 |
| h29 | WPM | 3.63±0.01 | 4.54±0.02 | 1.93±0.00 | 152.32±8.32 | 29.49±0.13 | 26.87±0.06 |
| h30 | WPM | 3.71±0.01 | 2.80±0.00 | 2.59±0.02 | 209.10±3.76 | 32.69±0.07 | 29.77±0.15 |
| p1 | M* | 3.35±0.02 | 6.69±0.02 | 1.19±0.02 | 445.91±5.30 | 16.67±0.11 | 33.73±0.23 |
| p2 | M | 4.43±0.03 | 4.74±0.02 | 0.89±0.02 | 666.07±1.34 | 23.42±0.00 | 51.81±0.02 |
| p3 | M | 3.81±0.01 | 10.29±0.05 | 1.44±0.02 | 844.02±11.53 | 18.57±0.37 | 51.68±0.03 |
| p4 | M | 3.73±0.01 | 10.58±0.00 | 1.90±0.04 | 1133.96±11.55 | 12.10±0.06 | 51.79±0.15 |
| p5 | M | 3.43±0.01 | 6.80±0.04 | 1.88±0.04 | 218.40±6.25 | 20.91±0.21 | 33.48±0.32 |
| p6 | M* | 4.31±0.01 | 2.93±0.00 | 0.68±0.04 | 853.22±3.78 | 12.28±0.08 | 48.80±0.03 |
| p7 | M | 3.59±0.00 | 7.24±0.02 | 2.11±0.02 | 830.33±3.66 | 18.27±0.04 | 48.06±0.15 |
| p8 | M | 4.26±0.00 | 5.13±0.00 | 1.83±0.00 | 854.88±3.60 | 23.99±0.03 | 48.89±0.00 |
| p9 | M* | 4.67±0.02 | 3.70±0.00 | 1.18±0.00 | 823.54±0.26 | 28.85±0.04 | 46.84±0.06 |
| p10 | M* | 4.15±0.02 | 3.64±0.02 | 0.81±0.02 | 645.13±6.25 | 20.55±0.16 | 49.53±0.05 |
| p11 | M | 3.73±0.01 | 5.53±0.04 | 1.90±0.00 | 878.12±6.89 | 20.11±0.17 | 51.25±0.41 |
| p12 | M | 4.07±0.02 | 3.28±0.00 | 0.90±0.07 | 367.71±4.30 | 27.18±0.14 | 48.70±0.38 |
| p13 | M | 4.11±0.01 | 16.66±0.02 | 3.33±0.04 | 898.59±12.97 | 18.82±0.00 | 59.72±0.30 |
| p14 | M | 3.27±0.01 | 4.31±0.02 | 0.30±0.00 | 67.91±0.41 | 22.70±0.03 | 27.50±0.09 |
| p15 | M | 3.59±0.01 | 6.30±0.00 | 0.29±0.02 | 564.73±1.98 | 13.43±0.01 | 35.79±0.00 |
| p16 | M* | 3.79±0.01 | 11.20±0.04 | 1.25±0.04 | 1257.30±0.93 | 9.06±0.09 | 49.14±0.07 |
| p17 | M | 3.47±0.01 | 4.86±0.02 | 0.28±0.04 | 359.04±3.85 | 13.39±0.07 | 42.35±0.01 |
| p18 | M* | 3.35±0.01 | 6.83±0.04 | 0.59±0.02 | 222.24±2.53 | 10.97±0.06 | 36.23±0.08 |
| p19 | M | 3.37±0.01 | 4.11±0.02 | 0.48±0.00 | 327.08±3.94 | 11.57±0.09 | 36.26±0.03 |
| p20 | M* | 3.92±0.01 | 8.26±0.05 | 1.01±0.05 | 1188.18±6.02 | 15.93±0.03 | 43.55±0.01 |
| p21 | M | 3.53±0.01 | 2.94±0.02 | 0.45±0.00 | 418.44±3.73 | 23.83±0.01 | 27.92±0.14 |
| p22 | M | 4.25±0.01 | 7.30±0.04 | 1.86±0.12 | 1079.16±4.71 | 22.99±0.00 | 44.19±0.16 |
| p23 | M | 3.74±0.02 | 6.20±0.00 | 2.40±0.07 | 622.80±5.94 | 24.79±0.11 | 39.45±0.03 |
| p24 | M | 3.42±0.02 | 17.00±0.00 | 3.80±0.00 | 509.80±7.90 | 28.17±0.44 | 32.19±0.13 |
| p26 | M | 4.23±0.01 | 7.99±0.02 | 1.15±0.04 | 1125.03±2.97 | 9.53±0.02 | 46.96±0.18 |
| p27 | M | 3.91±0.01 | 3.68±0.04 | 1.33±0.07 | 381.88±6.22 | 25.25±0.16 | 43.61±0.15 |
| p28 | M | 3.71±0.01 | 7.06±0.02 | 0.78±0.04 | 585.19±10.27 | 18.94±0.33 | 47.71±0.11 |
| p29 | M | 3.92±0.01 | 5.05±0.00 | 0.83±0.04 | 739.92±8.92 | 20.83±0.09 | 49.97±0.17 |
| P30 | M | 3.94±0.01 | 4.83±0.00 | 0.96±0.02 | 522.75±8.12 | 19.64±0.01 | 46.02±0.27 |

aa: amino acids. Ala: alanine. RPM: red proso millet. WPM: white proso millet. M: millet.

* Samples fermented from the raw material containing millet and a small amount of rice (<10%).
