# Supplementary figures and images for: Microbial Diversity and Biochemical Analysis of Suanzhou: A Traditional Chinese Fermented Cereal Gruel
Source: Front Microbiol. 2016 Aug 25;7:1311. doi: 10.3389/fmicb.2016.01311 (PMC4997791; doi:10.3389/fmicb.2016.01311)

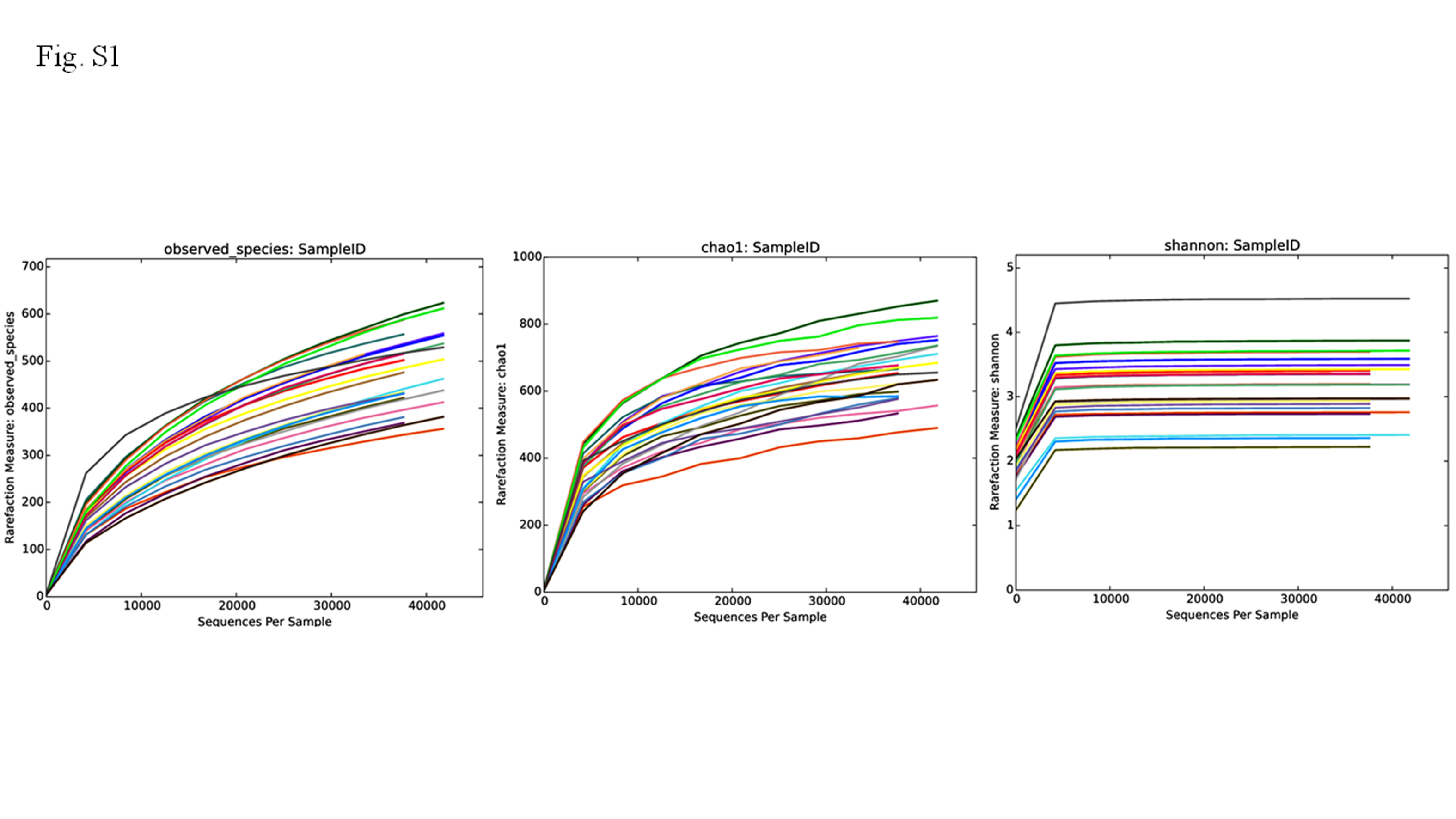

Supplement: Figure S1 — The collector retraction curves of the observed species, Chao1, and Shannon indice of the metagenomic libraries of the 24 Suanzhou samples. [file Image1.tif]

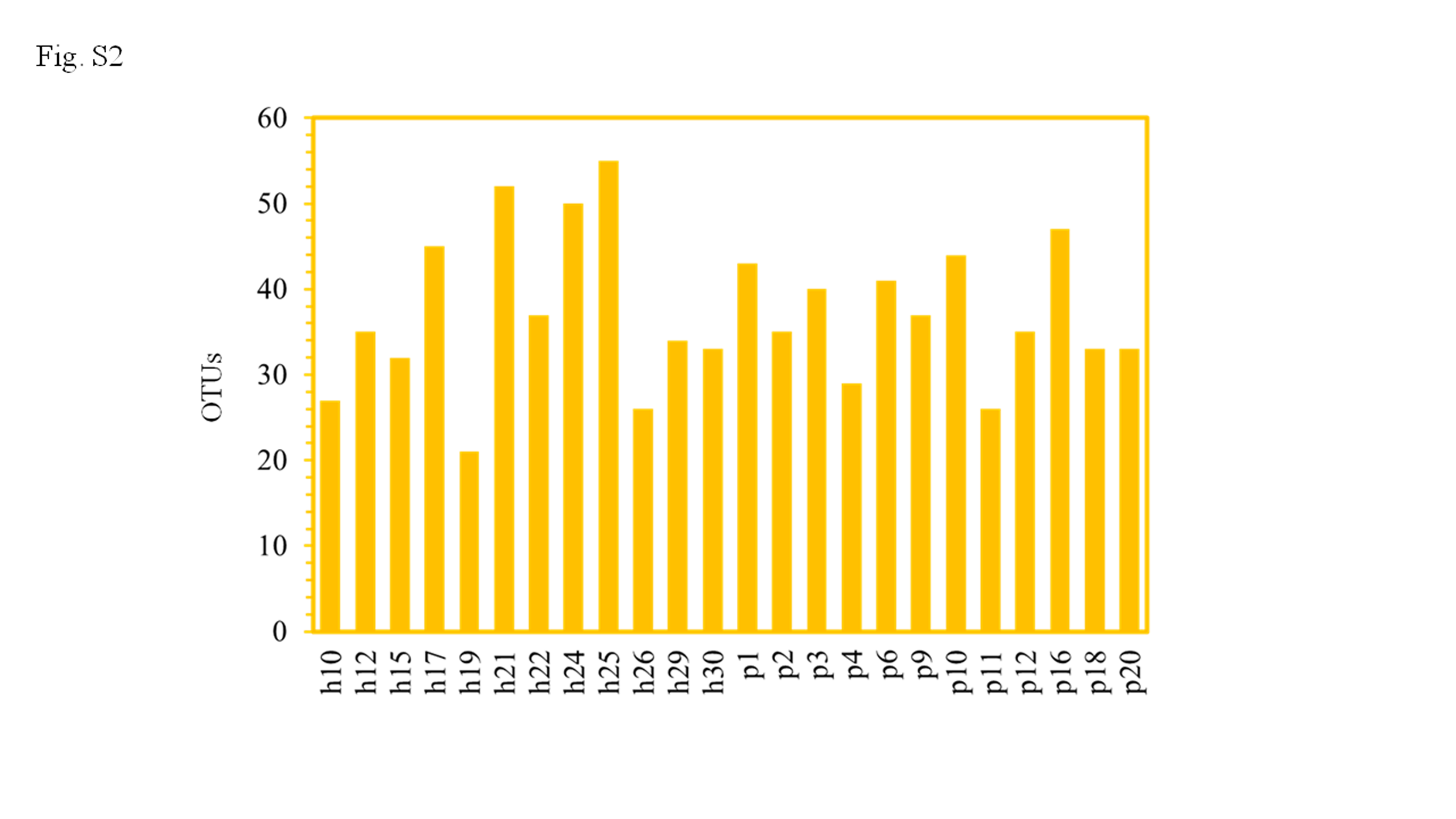

Supplement: Figure S2 — The OTUs distributions in the 24 Suanzhou samples. [file Image2.tif]

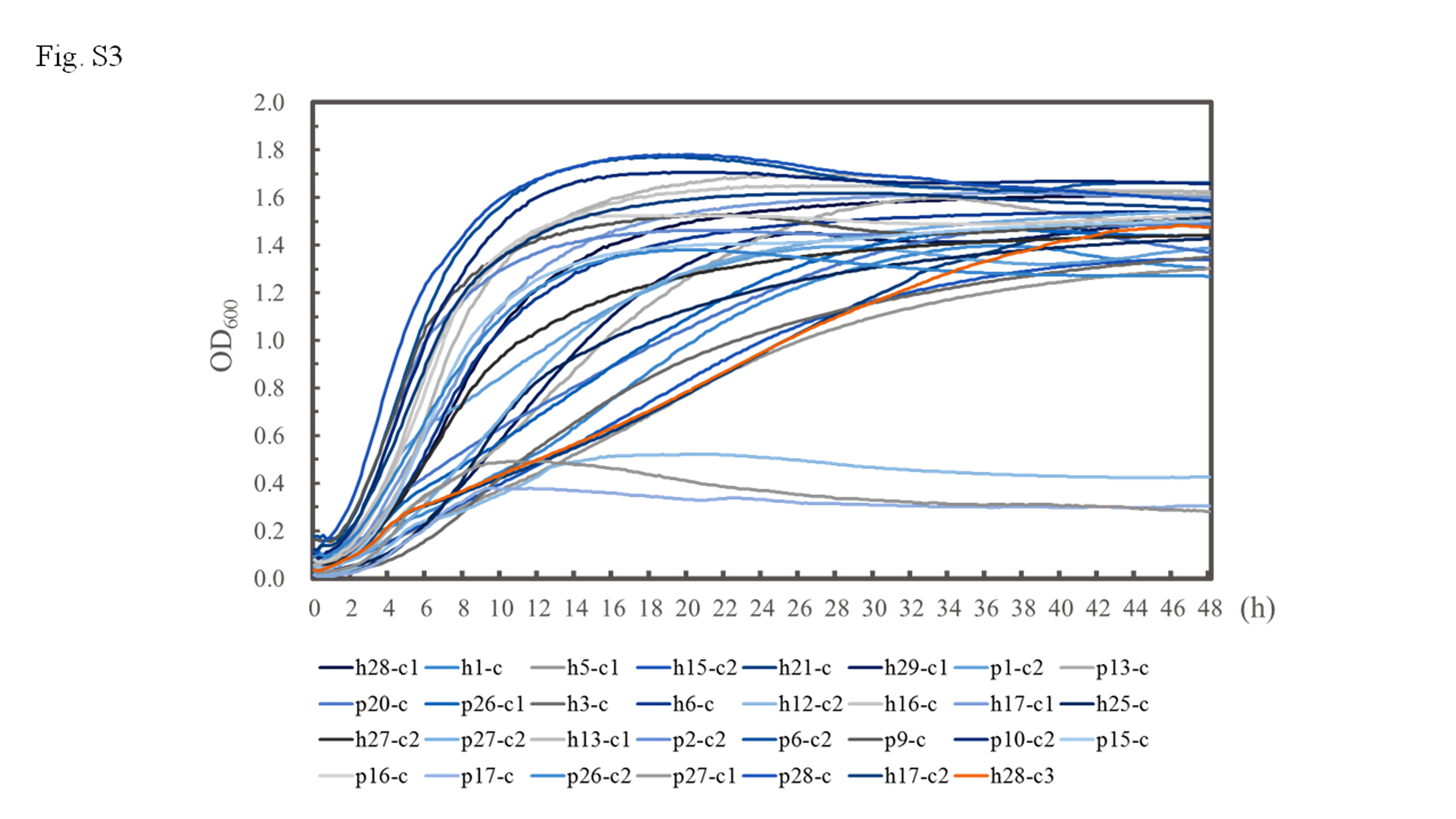

Supplement: Figure S3 — Growth curves of the isolated lactic acid bacteria. [file Image3.tif]
